# Pathomic model to predict the expression of UQCRH and overall survival of lung adenocarcinoma patients

**DOI:** 10.1186/s13000-026-01770-2

**Published:** 2026-02-13

**Authors:** Yong Chen, Jie Liu, Guoping Li, Huifang Huang

**Affiliations:** 1 Central Laboratory, Fujian Medical University Union Hospital, Fuzhou, 350009 China; 2 Department of Laboratory Medicine, Fuzhou First General Hospital Affiliated with Fujian Medical University, Fuzhou, 350005 China; 3 Department of Pathology, The First Affiliated Hospital of Fujian Medical University, Fuzhou, 350001 China

**Keywords:** Pathomics, UQCRH, Machine learning, Lung adenocarcinoma, Prognostic analysis

## Abstract

**Background:**

Ubiquinol-cytochrome c reductase hinge protein (UQCRH) is a component of mitochondrial respiratory chain complex CIII. Its relationship with human cancer has been less studied. Pathomics uses artificial intelligence algorithms to collect histopathological image features and perform joint analysis by combining gene and transcriptome data. In this study, a pathomics prediction model was established based on UQCRH expression and histopathological images of lung adenocarcinoma (LUAD). Prognostic value and other analyses were conducted based on this model.

**Methods:**

The expression level of UQCRH in 33 types of human cancers was measured. Its relationship with the survival of the primary LUAD samples with complete pathological images, gene expression data, and clinical information were divided into high and low expression groups based on the expression level threshold of the UQCRH gene (Table S1). LUAD patients was studied. Pathomic prediction model was established by using machine learning algorithms according to the UQCRH expression level and the characteristics of LUAD histopathological images. Based on this prediction model, survival analysis, molecular pathways, immune infiltration, immunological subtypes, ICI treatment prediction, and drug sensitivity analyses were performed.

**Results:**

UQCRH is highly expressed in various cancers, including LUAD. In addition, we verified that UQCRH is overexpressed in human LUAD tissues. High expression of UQCRH is worse prognostic factor for LUAD patients. A pathomic prediction model was constructed based on the UQCRH expression level and histopathology image features. The pathomic score showed good correlation with the UQCRH expression level. Patients in the high-risk group of the pathomic prediction model had worse prognosis and higher tumor proliferation ability, but may have better response to immune checkpoint inhibitors (ICIs) therapy.

**Conclusion:**

We have established a pathomic prediction model for LUAD based on gene expression values and according to histopathological image features, which can predict patient survival prognosis and has potential guiding value for ICIs therapy.

**Graphical Abstract:**

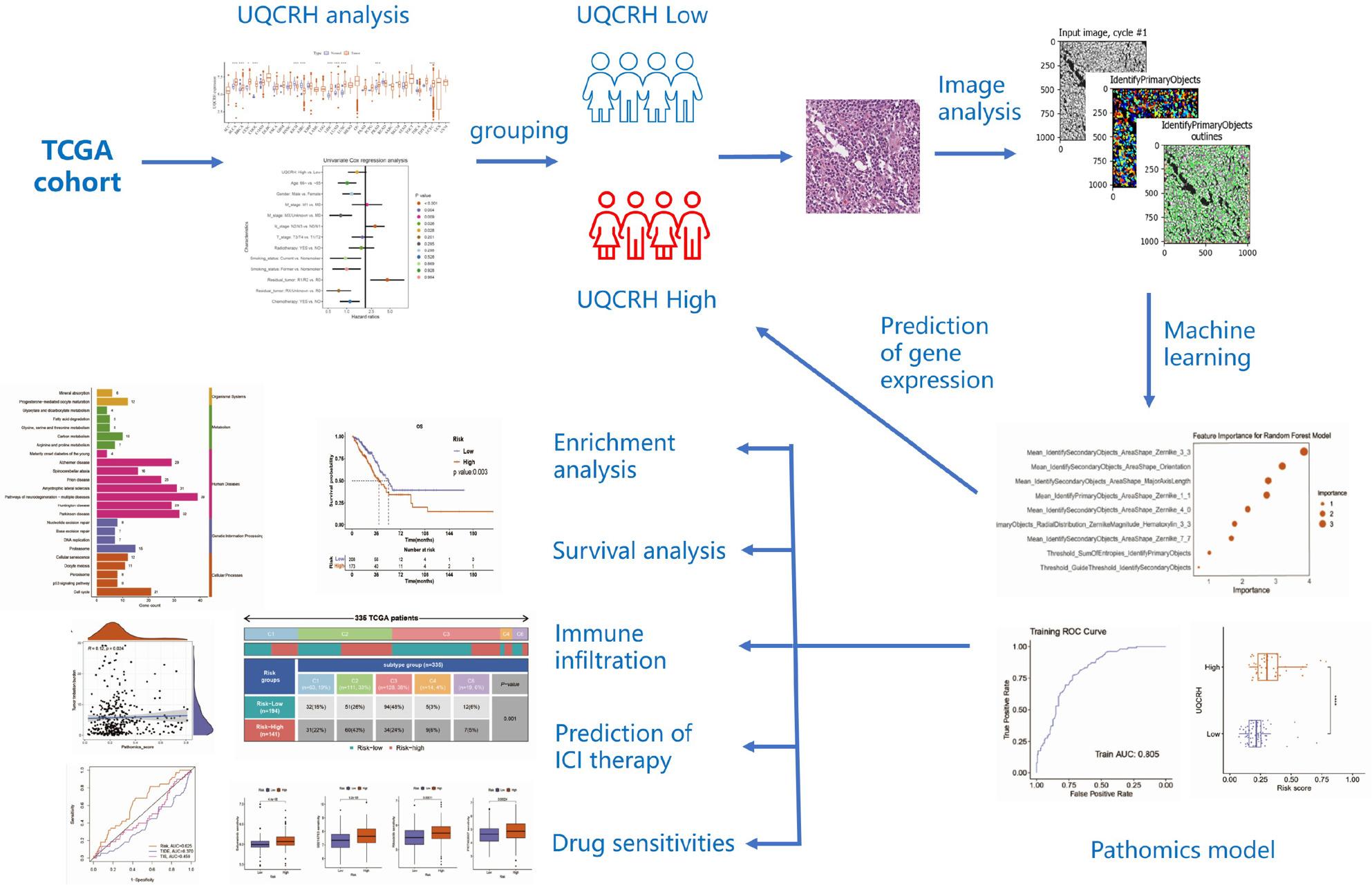

**Supplementary Information:**

The online version contains supplementary material available at 10.1186/s13000-026-01770-2.

## Introduction

Lung cancer is the most common and deadly cancer worldwide, with over 2.1 million new cases and more than 1.8 million deaths annually [1]. Approximately 80% of lung cancers are non-small cell lung cancer (NSCLC), which mainly includes two histological subtypes, lung adenocarcinoma (LUAD) and lung squamous cell carcinoma (LUSC), with LUAD being the predominant type. In recent years, the use of small molecule inhibitors and immune checkpoint inhibitors has led to a modest improvement in the 5-year survival rate for lung cancer patients. However, compared to LUSC, LUAD tends to be more resistant to immune checkpoint inhibitor therapy and has a poorer prognosis [2]. Therefore, early identification of high-risk patients with poor prognosis and those who may develop resistance to ICI treatment is crucial for the management and treatment of lung cancer patients.

Ubiquinol-cytochrome c reductase hinge protein (UQCRH) is a subunit of the cytochrome bc1 complex in the mitochondrial respiratory chain, which is crucial for the formation of cytochrome c1 and cytochrome c complexes [3]. UQCRH is widely expressed in most tissues, especially in organs with active energy metabolism. Deletion of two-exon in UQCRH can lead to recurrent episodes of severe ketoacidosis, excessive blood ammonia, hypoglycemia, and neurological symptoms related to impaired function of mitochondrial respiratory chain complex CIII. Mice with UQCRH knockout have largely reproduced the symptoms observed in human patients and tend to die prematurely around 12 weeks [4]. However, few studies have reported on the association between UQCRH and human cancer. It was found that UQCRH is overexpressed in cancer tissues such as multiple myeloma, breast, prostate, brain, and bladder cancer [5, 6]. The UQCRH protein is also overexpressed in tumor tissues and serum of patients with lung adenocarcinoma [7]. However, the relationship between UQCRH and patient prognosis, immune infiltration, drug therapy, etc., as well as its potential as a diagnostic and prognostic indicator, has not yet been studied.

Artificial intelligence algorithms have made significant progress in the field of medicine, particularly in the diagnosis and treatment of cancer. Through machine learning and deep learning, analyzing tissue pathology images of tumors can extract rich information beneficial for precision medicine, improving the efficiency, accuracy, and consistency of tissue pathology assessments [8, 9]. In addition, through artificial intelligence methods, high-throughput features can be extracted from pathological images stained with hematoxylin and eosin (H&E), combined with pathological morphology information and genetic data for analysis to find new image features useful for prognosis, a method known as pathomics [10–12]. Pathomics is not only valuable in prognostic assessment of malignant tumors but also provides decision support for potential treatments [13, 14].

This study took the UQCRH gene as the entry point and established a prediction model for LUAD survival prognosis based on pathologic genomics risk scores by combining cancer transcriptomics and pathologic genomics research methods. Subsequently, survival analysis, molecular pathways, immune infiltration, immune subgroups, ICI treatment prediction, and drug sensitivity were analyzed based on this prediction model.

## Materials and methods

### Gene expression analysis and patient grouping

The RNA-seq data, clinical information, and tumor mutation data from TCGA were downloaded from the UCSC Xena database (https://xenabrowser.net/). The TIDE score files were obtained from the TIDE website (http://tide.dfci.harvard.edu/). The expression differences of UQCRH between normal and tumor tissues were analyzed using the Wilcoxon test. In the primary LUAD patient samples, the cutoff value for UQCRH expression level was determined using the R package “survminer” with a value of 6.381. Patient demographics and clinicopathological characteristics are summarized in Table S1. The data were randomly divided into training and validation cohorts in a ratio of 7:3. The pathological histology feature values in the training cohort were standardized using z-score normalization, and the validation cohort was normalized using the mean and standard deviation from the training cohort (The inclusion and exclusion criteria are shown in Table S2).

### Immunohistochemistry

To further validate the results in the public database, we examined the expression levels of UQCRH in 6 formalin-fixed paraffin-embedded (FFPE) primary lung adenocarcinoma specimens and adjacent non-cancerous tissue, collected from The Fuzhou First General Hospital in 2024. All lung adenocarcinoma cases were classified as the acinar subtype according to the latest World Health Organization(WHO) classification criteria. The procedure is briefly outlined as follows: First, the tissue slides were de-paraffinized with xylene, hydrated with graded ethanol, and treated with 1× citrate antigen repair solution at 100 °C for 30 min. Next, the slices were placed in 3% hydrogen peroxide for 15 min to inactivate endogenous peroxidase activity. Sections were blocked with 5% bovine serum albumin (BSA) for 30 min at room temperature, followed by overnight incubation at 4 °C with a rabbit monoclonal anti-UQCRH antibody (Abcam, cat# ab154803, 1:2000 dilution). Subsequently, the slides were incubated with the secondary antibody (Goat anti-Rabbit, Dako) at 37 °C for 1 h. Finally, antigen detection was performed using DAB solution, and the nuclei were stained with hematoxylin.

### Survival analysis

Survival analysis was performed on each variable using the R package “survival”. The R package “survminer” was used to summarize and visualize the results of the analysis. Kaplan-Meier survival curves were used to display the changes in survival rates across different groups. Log-rank tests were conducted to assess the significance of survival rates between different groups. Univariate Cox regression was used for comparative association analysis to explore the factors affecting OS, while multivariate Cox regression was used to investigate whether a factor is an independent predictor of OS, as well as to explore the effects of multiple factors. Forest plots were generated using the R package “forestplot”.

### Histopathological image processing

H&E-stained histopathological images (20× or 40× magnification) were obtained from TCGA (https://tcga-data.nci.nih.gov/tcga/)[11, 15]. The tissue regions in the pathological sections were obtained using the Otsu algorithm (https://opencv.org/)[16]. The 40× images were segmented into multiple sub-images of 1000 × 1000 pixels, while the 20× images were segmented into multiple sub-images of 500 × 500 pixels. Ten randomly selected sub-images from each pathological image were used for subsequent analysis [11, 15]. Cell nuclei segmentation and feature extraction were performed using CellProfiler. First, the HE images were deconvoluted using the “UnmixColors” module. Subsequently, the deconvolved images were automatically segmented using the “IdentifyPrimaryObjects” module and the “IdentifySecondaryObjects” module to identify cell nuclei and cytoplasm. Further, quantitative image features of object shape, size, texture, and pixel intensity distribution were extracted using multiple modules, including measurement modules for “Object Intensity Distribution”, “Object Intensity”, “Texture”, and “Object Size Shape“ [17, 18].

### mRMR_RFE feature selection and random forest model establishment

Feature selection was performed using the Max-relevance Min-redundancy (mRMR) and recursive feature elimination (RFE) algorithms. The mRMR method selected the top 20 features, which were then further screened using RFE [19–21]. The selected features were used to construct a model using the random forest algorithm on the training set [22]. The performance of the model was evaluated in both the training and validation sets. The evaluation metrics included accuracy (ACC), specificity (SPE), sensitivity (SEN), positive predictive value (PPV), and negative predictive value (NPV). The receiver operating characteristic curve (ROC) was plotted. Calibration of the prediction model was assessed by plotting the calibration curve and performing the Hosmer-Lemeshow goodness-of-fit test. The overall performance of the prediction model was quantified using the Brier score. The clinical benefit of the prediction model was demonstrated by plotting the decision curve (DCA).

### Pathomic score and prognostic analysis

The pathomic score of the intersecting samples was calculated using a pathomics model, which was then combined with clinical data. The cutoff value was calculated using the “survminer” package, and it was used to categorize the data into Low/High binary variables (risk). A baseline table of clinical variables was created based on the Low/High risk score grouping. The Kaplan-Meier survival curve was plotted using the “survival” package, and the Log-rank test was performed to assess the significance of survival rates between different groups. Univariate Cox regression was used to explore the factors affecting OS, while multivariate Cox regression was used to determine whether a factor is an independent predictor of OS or not, as well as to investigate the effect of multiple factors.

### Enrichment analysis

Using the R packages “limma” and “clusterProfiler”, we performed Gene Ontology (BP, CC, MF) and KEGG enrichment analysis on the differentially expressed genes between the high-risk and low-risk groups [23]. For BP, CC, and MF enrichment analysis, we visualized the top 10 significantly enriched pathways. For KEGG enrichment analysis, we visualized all significantly enriched pathways.

### Analysis of immune gene expression differences and immune cell infiltration

The differential expression of immune genes between the high-risk and low-risk groups was analyzed using the Wilcoxon test, and the genes with *p* < 0.05 were visualized. The gene expression matrix of lung adenocarcinoma samples was uploaded to the CIBERSORTx database (http://cibersortx.stanford.edu/) to calculate the immune cell infiltration for each sample. The degree of immune cell infiltration between the high and low expression groups was analyzed using the R package “limma”.

### Tumor mutation burden

Tumor mutation burden (TMB) is defined as the number of somatic mutations per megabase of the genomic sequence and has demonstrated potential as a predictive biomarker for identifying cancer patients most likely to respond to immune checkpoint inhibitors [24]. The maf format mutation data of TCGA-LUAD were downloaded from the TCGA database (https://portal.gdc.cancer.gov/). Spearman correlation analysis was used to examine the correlation between pathomic score and TMB.

### Comparison of multiple models

The expression matrix of TCGA-LUAD was uploaded to the TIDE database (http://tide.dfci.harvard.edu) to evaluate the response of immune checkpoint inhibitors (ICI) using the TIDE algorithm [25, 26]. The results of the website analysis were statistically analyzed and visualized using R language. The TIS algorithm calculates the TIS score as a linear combination of 18 algorithmic genes based on gene expression data from TCGA database for primary tumor cancer, with the formula TIS = $\:{\sum\:}_{i=1}^{18}\mathrm{x}i\mathrm{w}i$. Using the “timeROC” package, time-dependent ROC curves were plotted to illustrate the predictive ability of factors at different time points. Corresponding tROC curves were plotted at different time points (12 months, 18 months, and 24 months) after the diagnosis of lung adenocarcinoma to assess the discriminatory power of pathomic score, TIDE, and TIS in predicting patient survival at different time points.

### Drug sensitivity analysis

The IC50 values of 198 drugs were downloaded from the GDSC database (http://www.cancerrxgene.org/). Using the R package “oncoPredict”, the IC50 values for each sample were predicted based on RNA-seq data. The difference in drug IC50 values between the high-risk and low-risk groups was analyzed using the Wilcoxon test, and the results with *p* < 0.001 were visualized.

### Statistical analysis

The statistical analysis was performed using R software (version 4.2.0; https://www.Rproject.org). The expression levels of mRNA were calculated as mean ± SEM and compared between tumor tissues and normal tissues using the Wilcoxon signed-rank test. One-way ANOVA was used to compare multiple groups. Pearson correlation analysis was used to explore the correlation between continuous variables. Univariate COX proportional hazards regression model or log-rank test was used to evaluate the correlation between gene expression and overall survival rate of patients. *P* < 0.05 was considered statistically significant, with *p < 0.05, **p < 0.01, ***p < 0.001, and ****p < 0.0001.

## Results

### UQCRH is highly expressed in LUAD and is associated with poor prognosis

**Fig. 1:**
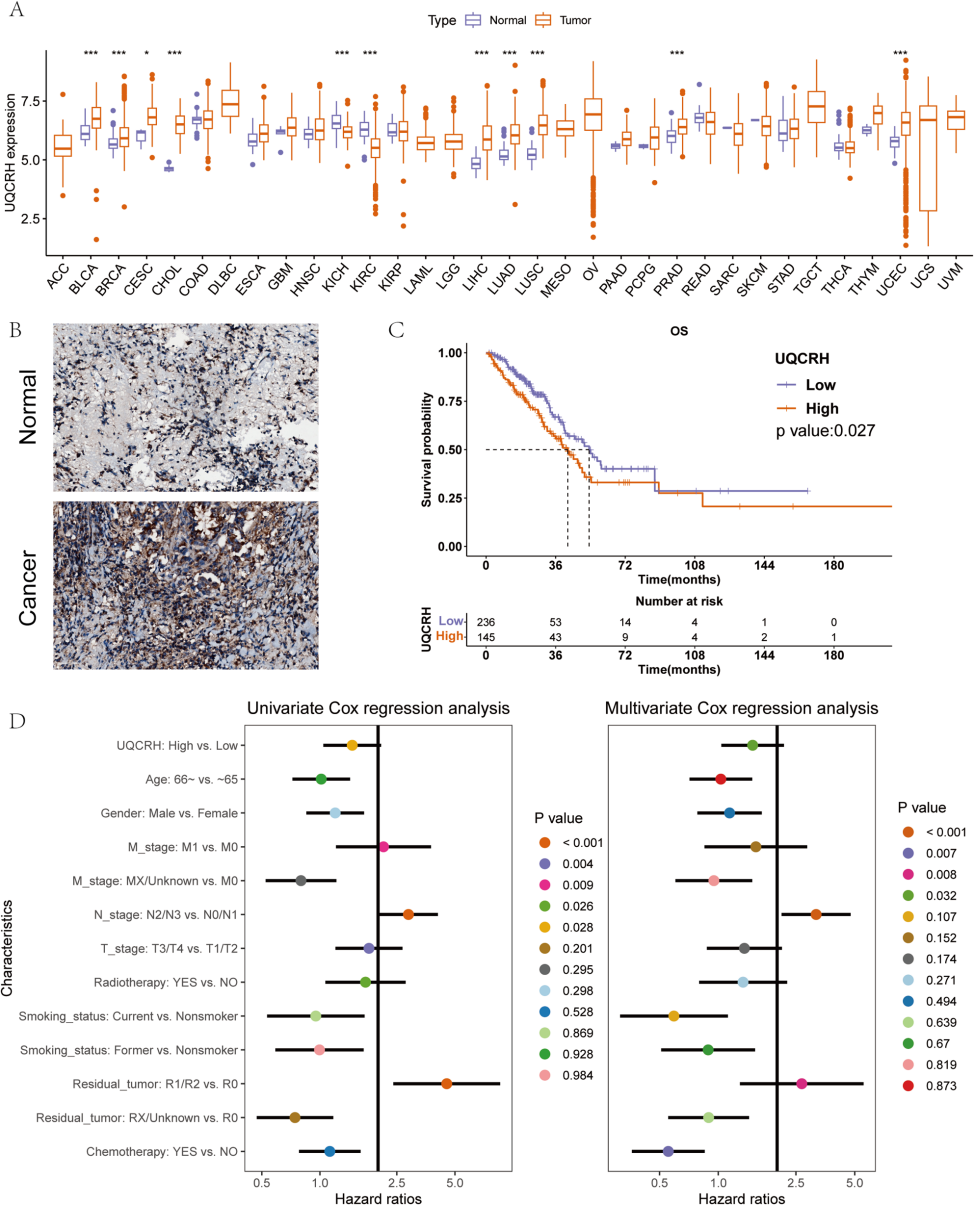
The mRNA expression and prognostic analysis of UQCRH. **A** The mRNA expressions of UQCRH in 33 types of human cancers; (**B**) Representative IHC images of UQCRH low and high expression in LUAD patients; (**C**) Kaplan-Meier curve for the prognostic analysis of UQCRH in LUAD; (**D**) Univariate (left) and multivariate (right) Cox regression analysis of the Pathomic feature model. *p < 0.05, **p < 0.01, and ***p < 0.001

We first analyzed the expression of UQCRH in 33 types of human cancers. UQCRH was upregulated across multiple cancers, including LUAD (Fig. 1A). To further validate the results from the public database, we performed immunohistochemical staining to assess the expression levels of UQCRH in cancerous and adjacent non-cancerous tissues from LUAD patients. UQCRH was highly expressed in tumor tissues but low in adjacent non-cancerous tissues, consistent with transcriptomic data (Fig. 1B). A total of 381 LUAD patients were selected according to the inclusion criteria, and were divided into a high expression group (*n* = 145) and a low expression group (*n* = 236) based on a cutoff value of UQCRH expression of 6.381(Table S1). As shown in Fig. 1C, high expression of UQCRH was associated with worse OS (*P* = 0.027), with a median survival time of 53.33 months in the low expression group and 42.27 months in the high expression group. Univariate Cox regression also showed that high expression of UQCRH was a worse prognostic factor for OS (HR = 1.67, 95%CI 1.182–2.358, *P* = 0.004), and multivariate Cox analysis results were similar (HR = 1.568, 95%CI 1.068–2.301, *P* = 0.022) (Fig. 1D).

### Analysis of LUAD pathology images and pathomic score

**Fig. 2:**
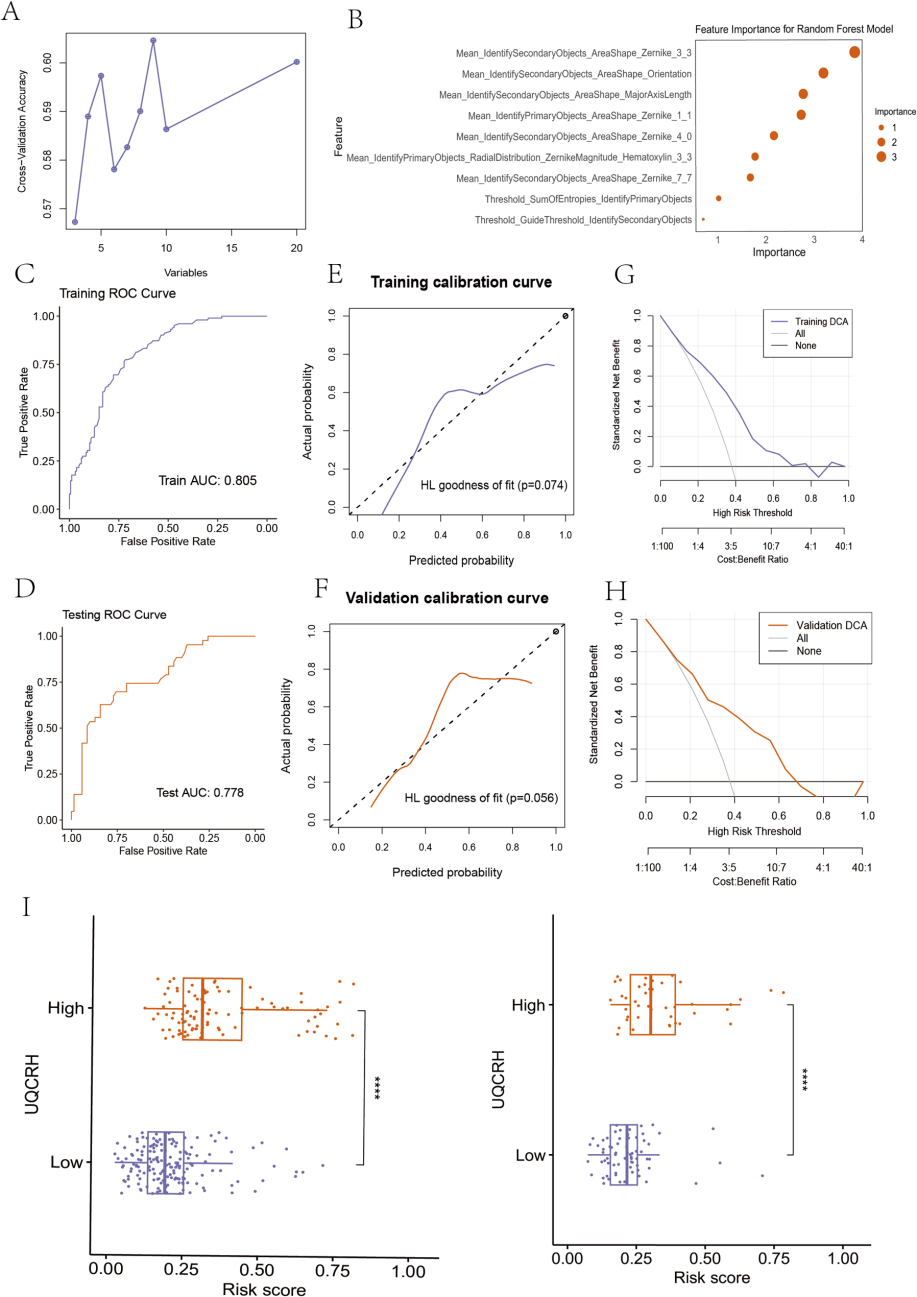
Pathomic feature model of LUAD. A Pathological feature selection; (B) Feature importance of the random forest model; C: Performance evaluation of the model in the (C) training set and (D) testing set; The calibration curves of the (E) training set and (F) testing set; The DCA curves of the (G) training set and (H) testing set; (I) The UQCRH mRNA expressions of the Pathomics scores high/low groups in the training (left) and testing set (right)

After segmentation and selection of quantitative features through image analysis algorithms, the best feature subset is screened using mRMR and RFE algorithms. As shown in Figs. 2A, 9 features are selected. The selected features are used to build a model in the training set through the random forest (RF) algorithm. Figure 2B shows the importance of the selected features in the random forest algorithm. The RF model demonstrated strong predictive performance. The AUC value of the model in the training set is 0.805; the validation set is 0.778 (Fig. 2C and D). Calibration curves and Hosmer-Lemeshow goodness-of-fit tests show that the pathological genomic prediction model has good consistency between the predicted probability of high gene expression and the true value (*P* > 0.05) (Fig. 2E and F). DCA shows that the model has high clinical practicality (Fig. 2G and H). The RF model prediction value is the Pathomic score. Figure 2I shows the difference in Pathomic score between the high and low expression groups of UQCRH gene in the training set (left) and validation set (right). The distribution of risk scores between the high and low expression groups of genes in both sets has significant differences.

### Higher pathomic score is associated with poor prognosis

**Fig. 3:**
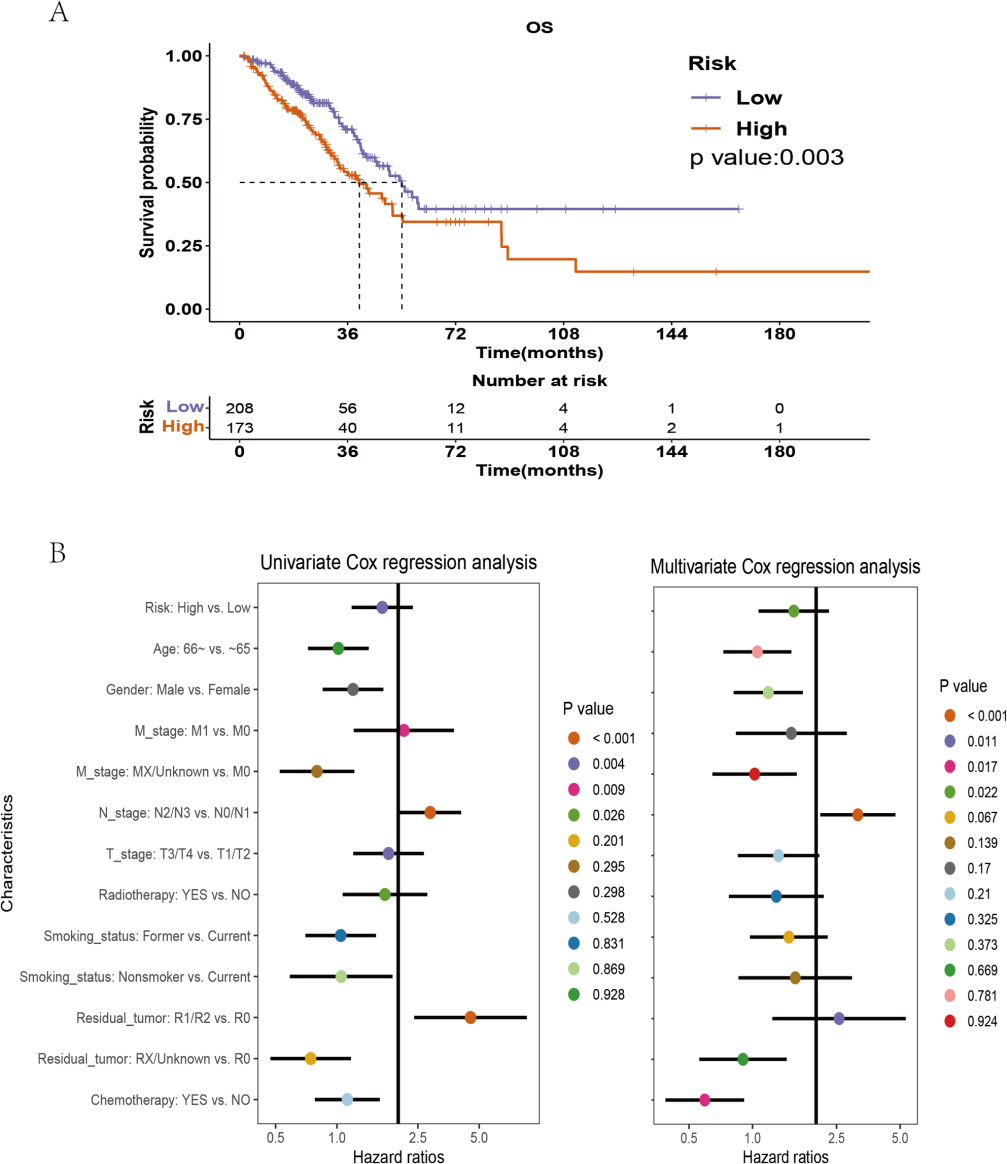
Prognostic analysis of LUAD based on pathomic feature model. A Kaplan-Meier curve for the pathomic feature model and OS; (B) Univariate (left) and multivariate (right) Cox regression analysis of the pathomic feature model

According to the Pathomic score cutoff of 0.246, patients are divided into high-risk and low-risk groups(Table S2). As shown in Fig. 3A, the median survival time of the high-risk group is 39.9 months, while that of the low-risk group is 54.07 months, indicating a significant correlation between a higher Pathomic score and worsening OS (*p* = 0.003). Similarly, univariate Cox analysis and multivariate analysis also show that a higher Pathomic score is an unfavorable factor for OS (Fig. 3B).

### Enrichment analysis of high and low risk groups in pathological genomics

**Fig. 4:**
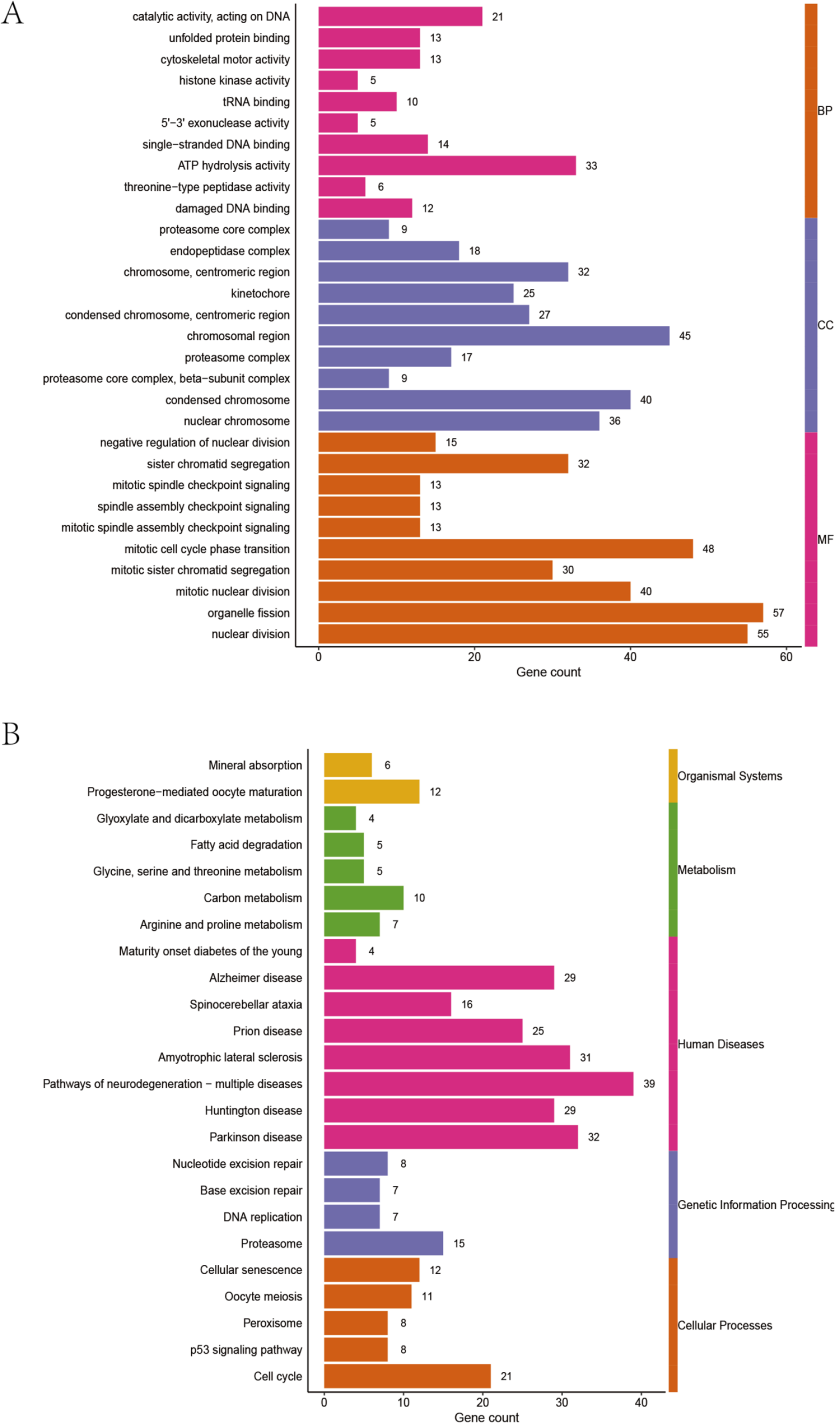
Enrichment analysis of DEGs in pathomic feature model groups. A GO enrichment; (B) KEGG enrichment

The enrichment analysis of the high and low risk groups shows that DEGs in the high and low risk groups are significantly enriched in related pathways such as cell mitosis, cell cycle, regulation of chromatin structure, and proteasome activity (Fig. 4A). KEGG enrichment analysis suggests that DEGs in the high and low risk groups are significantly enriched in signal pathways such as cell cycle, DNA replication, p53 signaling pathway, and DNA repair (Fig. 4B).

### Immune infiltration analysis

**Fig. 5:**
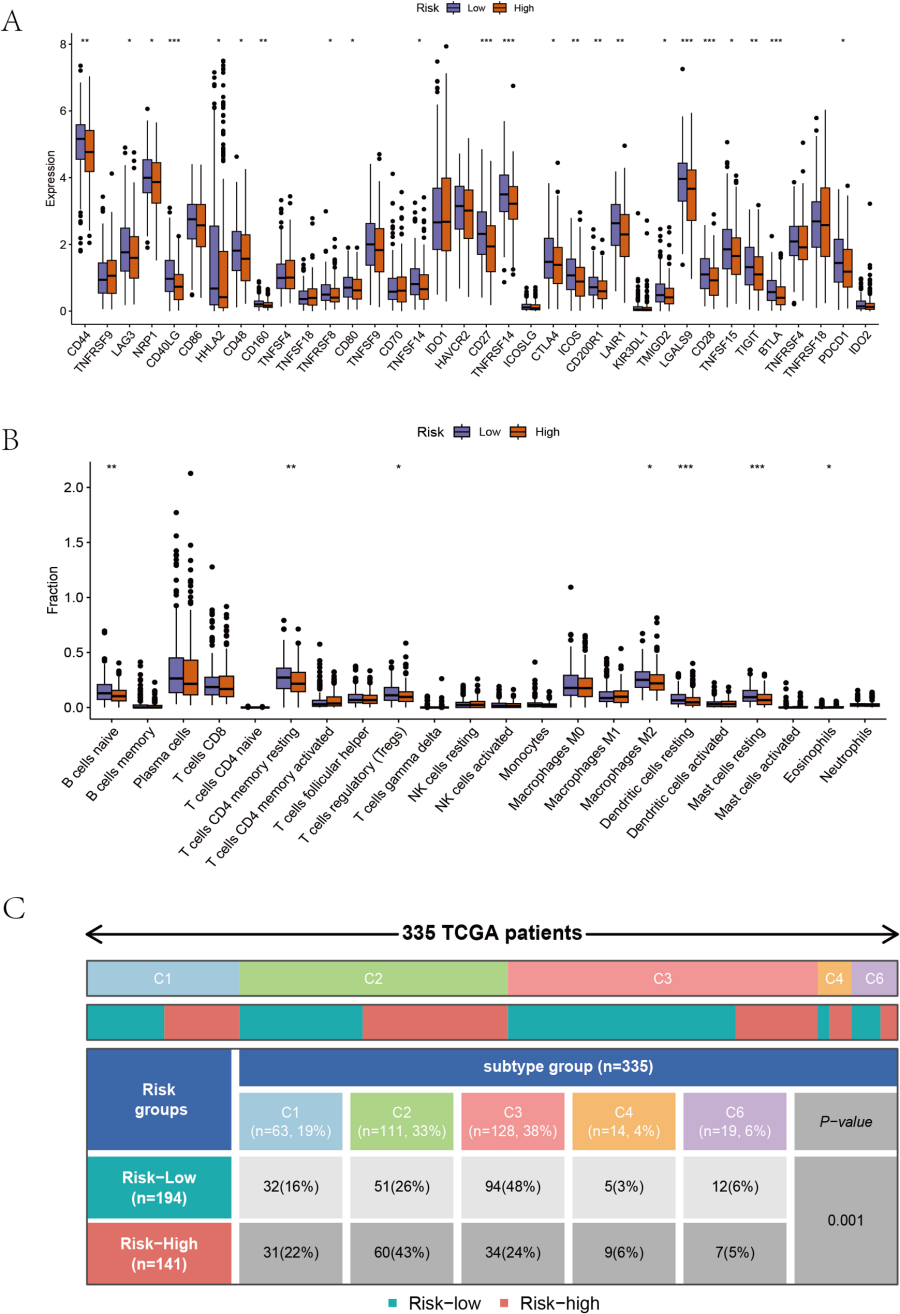
Immune functions analysis of Pathomic feature model groups. A Immune genes expressions in high/low groups; (B) Immune cells infiltrations in high/low groups; (C) Immune subtype of high/low groups

The differential gene expression analysis of immune genes shows that CTLA4, PDCD1, LAG3, etc. have significantly lower expression levels in the high-risk group (Fig. 5A). The situation of immune cell infiltration shows that T cell CD4 memory resting, macrophages M2, dendritic cells resting, mast cells resting, etc. have increased infiltration levels in the low-risk group, while there is no significant difference in T cells CD8 infiltration levels between the two groups (Fig. 5B). The immune subtype analysis shows that immune subtype C2 is higher in the high-risk group, while subtype C3 is lower compared to the low-risk group, showing significant differences (Fig. 5C).

### Predictive analysis of ICI therapy

**Fig. 6:**
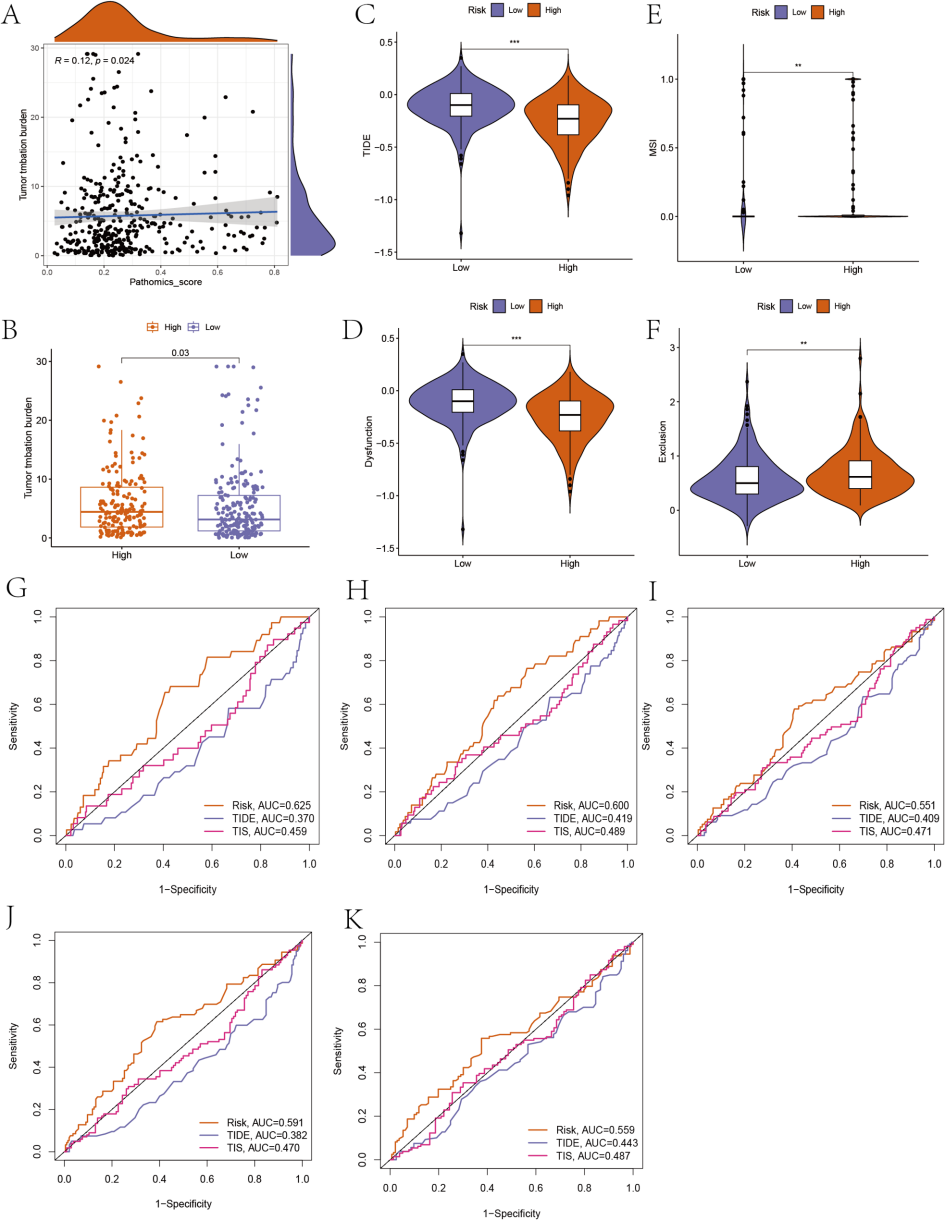
Prediction of ICIs therapy response of pathomic feature model groups. A The correlation between TMB and Pathomics scores; (B) TMBs of high/low groups; (C) The TIDE, (D) T cell dysfunction, (E) MSI; and (F) T cell exclusion scores of high/low groups; (G-K) the time ROC curves of 12, 18, 24, 30, and 36 months based on Pathomics scores, TIDE and TIS

Tumor mutation burden can be used to identify cancer patients most likely to respond to immune checkpoint inhibitors. We analyzed the correlation between Pathomic score and TMB, and the results showed that Pathomic score was positively correlated with TMB, with a correlation coefficient of 0.12 (Fig. 6A and B). TIDE score can better evaluate the efficacy of anti-PD-1 and anti-CTLA4 therapies. As shown in Fig. 6C, the low-risk group of Pathomic score had higher TIDE (Fig. 6C) and T cell dysfunction (Fig. 6D), while microsatellite instability (MSI) (Fig. 6E) and T cell exclusion (Fig. 6F) were lower. We calculated the TIS gene expression score based on TCGA expression data for LUAD patients and plotted time ROC curves based on TIDE and TIS for 12, 18, 24, 30, and 36 months, comparing them with our constructed Pathomic score. The results showed that at the 12-month time point, the predictive ability of Pathomic score was significantly better than that of TIDE and TIS (Fig. 6G-K).

### Drug sensitivity analysis

**Fig. 7:**
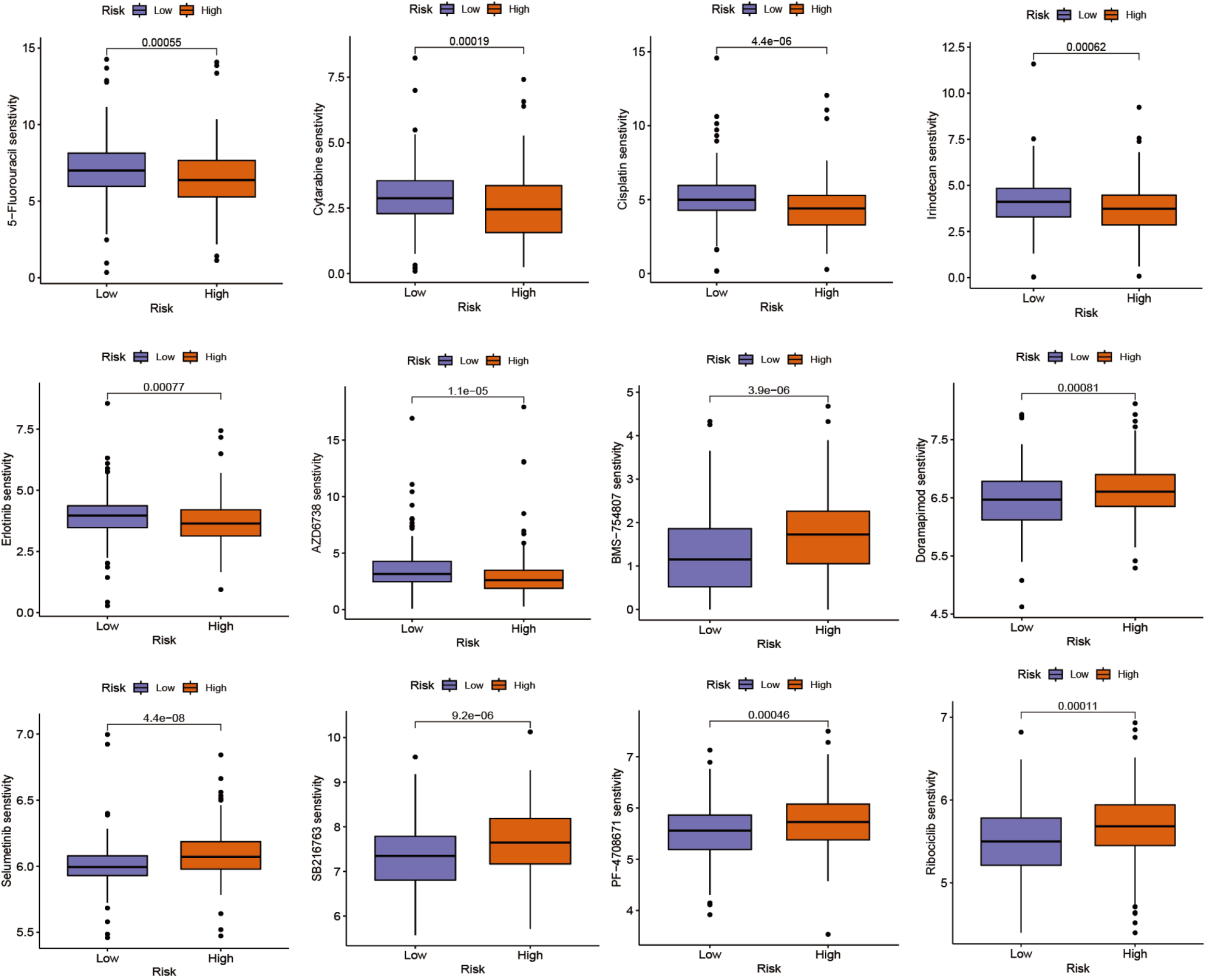
Drug sensitivities analysis of Pathomic feature model groups

We used the R package “oncoPredict” to predict the sensitivity of high- and low-risk groups to 198 drugs. There were significant differences in drug sensitivity between the two groups. As shown in Fig. 7, the high-risk group was more sensitive to 5-FU, cytarabine, cisplatin, erlotinib, irinotecan, and AZD6738, while the low-risk group was more sensitive to BMS-754,807, doramapimod, ribociclib, SB216763, selumetinib, and PF-4,708,671.

## Discussion

The mitochondrial membrane respiratory chain complex plays a crucial role in cellular energy metabolism and ATP synthesis, and its functional inhibition can lead to cell death. UQCRH is a key component of respiratory chain complex III, which collaborates with multiple proteins such as UQCRC2 and UQCRB to transfer electrons from ubiquinone to cytochrome c [3]. Previous research on UQCRH in cancer has mainly focused on expression and prognosis studies. Park et al. found that UQCRH mRNA is highly expressed in hepatocellular carcinoma and is associated with poor prognosis, tumor size, and poor differentiation [5]. Gao et al. discovered that the level of UQCRH in the serum of lung cancer patients increases and can serve as a diagnostic indicator for lung cancer [7]. Our pan-cancer analysis showed that UQCRH is upregulated in breast cancer, liver cancer, and lung cancer, and is associated with poor prognosis in LUAD, which is consistent with the results of these two studies.

Artificial intelligence has made significant progress in the recognition of pathological images [8, 27, 28]. Traditional pathology diagnosis mainly relies on the observation and analysis of doctors, which is susceptible to subjective factors. In recent years, many research teams have developed pathological image recognition systems based on deep learning, such as convolutional neural networks (CNN) and recurrent neural networks (RNN) [8]. These systems can automatically extract features from pathological images and learn through training data, enabling accurate identification of different types of tumors, inflammation, and other pathological changes. By grouping pathological images based on image features, it is also possible to combine with survival data for prognosis prediction.

One of the questions this study aims to explore is whether the expression values of specific genes can be associated with tissue histopathology image features. In this way, tissue histopathology image scores can represent the expression levels of specific genes or gene sets. We divided patients into two groups based on UQCRH expression levels and used machine learning and artificial intelligence algorithms to find differential features and establish a random forest model. The results showed that there is a good correlation between the pathomic score given by the model and gene expression values.

We used the pathomic score to evaluate the prognosis risk and predict the outcome of LUAD patients. The results showed that the model we constructed performed well in both the training set and validation set. The AUC value of the ROC curve indicates that the model can provide excellent evaluation for the prognosis of LUAD patients. In addition, we divided samples into high-risk and low-risk groups based on the median pathomic score of the model. Kaplan-Meier curves and Cox regression analysis showed that the model can be used for predicting the prognosis of LUAD patients. The construction and validation of this model provide feasible and effective evidence for the application of image learning models in LUAD prognosis assessment.

In image feature recognition algorithms, traditional statistics (TS), machine learning (ML), and deep learning (DL) are three commonly used methods [29]. The random forest algorithm is an ML algorithm. Compared to TS and DL, ML is very suitable for handling high-dimensional data and nonlinear relationships, with high accuracy and easy-to-understand models. ML algorithms have been widely used in medical imaging image analysis, for constructing radiomics prognostic models and predicting gene mutations [30–33]. This study shows the applicability of ML algorithms in tissue pathology image analysis.

We analyzed the enrichment of differentially expressed genes in GO and KEGG pathways in the high-risk and low-risk groups. We found that the differentially expressed genes were significantly enriched in pathways such as cell cycle, chromatin structure regulation, DNA replication, p53 signaling pathway, and proteasome activity. These pathways are related to malignant phenotypes of tumor cells, indicating that there is a clear distinction between high and low Pathomic score groups in terms of tumor malignancy.

Through the analysis of genomic and transcriptomic data from the TCGA database, we have gained a deeper understanding of the tumor microenvironment (TME). By examining the distribution and relationships between malignant cells, immune cells, and stromal cells, the immune status of cancer tissues has been classified into six distinct immunological subtype clusters: C1 (Wound Healing), C2 (IFN-γ Dominant), C3 (Inflammatory), C4 (Lymphocyte Depleted), C5 (Immunologically Quiet), and C6 (TGF-β Dominant) [34]. Among these subtypes, C4 and C6 have the worst prognosis. In our constructed pathomics score model, high-risk groups are more concentrated in the C2 subtype, while low-risk groups are more concentrated in the C3 subtype. Cancer tissues of the C2 subtype exhibit faster proliferation rates, higher tumor heterogeneity, and lower Th1:Th2 ratios, indicating a higher degree of malignancy.

Cancer tissues of the C2 subtype exhibit the highest M1/M2 macrophage ratio and strong CD8 signal, indicating that the high-risk group’s cancerous tissue may be rich in pro-inflammatory M1 macrophages and CD8 + T cells. Cancer tissues of the C3 subtype have slower proliferation rate, lower proportion of cancer cells, and relatively higher levels of Th17 and Th1 type T cells, displaying the highest level of immune cell infiltration among the six tumor subtypes. Therefore, from the perspective of immune subtype analysis, the malignancy level of tumor cells in the high-risk group is higher, which aligns with the differential gene enrichment observed in the enrichment analysis for the high-risk group. Since both high and low-risk groups show some degree of immune cell activity, solely based on immune subtype analysis, it is not yet possible to determine the difference in immunotherapy outcomes between the two groups.

TMB, TIDE, and TIS are all capable of predicting the efficacy of immune checkpoint inhibitors (ICI) therapy. Our findings indicate a positive correlation between Pathomics score and TMB. TIDE analysis demonstrates that the high-risk group has lower TIDE scores, which is consistent with the conclusions drawn from TMB analysis. Integrating comprehensive immunogenomic subtype analysis, TMB, and TIDE, we seem to be able to predict that the high-risk group may benefit more from ICI therapy. We plotted time ROC curves to evaluate the predictive performance of our pathomic score for ICI treatment outcomes, and the results showed that our Pathomic score had better predictive performance than TIDE and TIS.

We analyzed the sensitivity of high and low-risk groups to different chemotherapeutic agents and targeted therapeutic drugs. The differential drug sensitivities revealed significant disparities between the high and low-risk groups in terms of tumor cell characteristics and molecular features. The high-risk group was more sensitive to conventional chemotherapeutic agents, such as pyrimidine nucleoside synthesis inhibitors like 5-FU and cytarabine [35, 36], platinum agents like cisplatin, and DNA topoisomerase inhibitors like irinotecan [37]. In contrast, the low-risk group showed greater sensitivity to targeted agents acting on the receptor tyrosine kinase signaling pathway, including insulin-like growth factor receptor inhibitor BMS-754,807 [38], MEK1/2 inhibitor selumetinib [39, 40], and MAPK inhibitor doramapimod [41]. Based on these findings, we speculate that a combination therapy using conventional chemotherapeutic drugs and ICIs might yield better treatment outcomes for patients in the high-risk group.

The limitations of this study mainly lie in two aspects. Firstly, we only used the pathological images and gene expression data from the TCGA dataset, which has a relatively small sample size. Secondly, we did not employ multiple machine learning algorithms to analyze image features and compare their advantages and disadvantages. In future research, we will incorporate more external datasets or use self-collected data for support.

In summary, this study innovatively established a pathomics score prediction model based on image features, which can accurately predict the UQCRH gene expression levels in LUAD patients. Furthermore, the model based on the pathomics score can predict the prognosis of LUAD patients and has certain predictive and guiding significance for ICI treatment in LUAD patients.

## Supplementary Information

Supplementary Material 1.

Supplementary Material 2.

## Data Availability

Publicly available datasets were analyzed in this study. This data can be found here: the RNAseq data, clinical information, and tumor mutation data from TCGA were downloaded from the UCSC Xena database (https://xenabrowser.net/datapages/) and the H&E-stained histopathological images were downloaded from TCGA (https://tcga-data.nci.nih.gov/tcga/).
